# Mutations in the *MET* tyrosine kinase domain and resistance to tyrosine kinase inhibitors in non-small-cell lung cancer

**DOI:** 10.1186/s12931-023-02329-1

**Published:** 2023-01-25

**Authors:** Yu Yao, Huaping Yang, Bo Zhu, Song Wang, Jiaohui Pang, Xiaoying Wu, Yang Xu, Junli Zhang, Jinfeng Zhang, Qiuxiang Ou, Hui Tian, Zheng Zhao

**Affiliations:** 1 Department of Medical Oncology, First Affiliated Hospital of Xi’an Jiaotong University, Xi’an, 710049 Shaanxi China; 2 Department of Respiratory Medicine, Xiangya Hospital, Central South University, Changsha, 410008 Hunan China; 3 Department of Respiratory and Critical Care Medicine, First Affiliated Hospital of Xi’an Jiaotong University, Xi’an, 710049 Shaanxi China; 4 Geneseeq Research Institute, Nanjing Geneseeq Technology Inc., Nanjing, 210000 Jiangsu China; 5 Department of Thoracic Surgery, Ningbo Medical Centre Lihuili Hospital, Ningbo, 315046 China; 6 Shaanxi Cancer Hospital, 309 Yanta West Road, Xi’an, 710000 Shaanxi China

**Keywords:** *MET*, Tyrosine kinase domain, NSCLC, EGFR-TKI, Genomic profiling

## Abstract

**Background:**

The *Mesenchymal epithelial transition factor* (*MET*) gene encodes a receptor tyrosine kinase with pleiotropic functions in cancer. *MET* exon 14 skipping alterations and high-level *MET* amplification are oncogenic and targetable genetic changes in patients with non-small-cell lung cancer (NSCLC). Resistance to tyrosine kinase inhibitors (TKIs) has been a major challenge for targeted therapies that impairs their clinical efficacies.

**Methods:**

Eighty-six NSCLC patients were categorized into three cohorts based on the time of detecting *MET* tyrosine kinase domain (TKD) mutations (cohort 1: at baseline; cohort 2: after MET-TKI treatment; cohort 3: after EGFR-TKI treatment). Baseline and paired TKI treatment samples were analyzed by targeted next-generation sequencing.

**Results:**

*MET* TKD mutations were highly prevalent in *METex14*-positive NSCLC patients after MET-TKI treatment, including L1195V, D1228N/H/Y/E, Y1230C/H/N/S, and a double-mutant within codons D1228 and M1229. Missense mutations in *MET* TKD were also identified at baseline and in post-EGFR-TKI treatment samples, which showed different distribution patterns than those in post-MET-TKI treatment samples. Remarkably, H1094Y and L1195F, absent from MET-TKI-treated patients, were the predominant type of *MET* TKD mutations in patients after EGFR-TKI treatment. D1228H, which was not found in treatment-naïve patients, also accounted for 14.3% of all *MET* TKD mutations in EGFR-TKI-treated samples. Two patients with baseline *EGFR*-sensitizing mutations who acquired *MET*-V1092I or *MET*-H1094Y after first-line EGFR-TKI treatment experienced an overall improvement in their clinical symptoms, followed by targeted therapy with MET-TKIs.

**Conclusions:**

*MET* TKD mutations were identified in both baseline and patients treated with TKIs. *MET*-H1094Y might play an oncogenic role in NSCLC and may confer acquired resistance to EGFR-TKIs. Preliminary data indicates that *EGFR*-mutated NSCLC patients who acquired *MET*-V1092I or *MET*-H1094Y may benefit from combinatorial therapy with EGFR-TKI and MET-TKI, providing insights into personalized medical treatment.

**Supplementary Information:**

The online version contains supplementary material available at 10.1186/s12931-023-02329-1.

## Background

Lung cancer is the leading cause of cancer-related mortality worldwide, and non-small-cell lung cancer (NSCLC) represents the major histological subtypes of the disease [1]. A major challenge to improving the benefits of targeted therapies is understanding the molecular mechanisms of acquired resistance to tyrosine kinase inhibitors (TKIs).

Enormous efforts have been made to identify activating mutations, particularly those involved in cancer initiation and drug resistance. Approximately 3% of NSCLC patients harbor *MET* exon 14 skipping alterations (*METex14*), resulting in decreased MET turnover and extended oncogenic downstream signaling pathways [2, 3]. The focal genomic amplification of *MET* (*METamp*) may also rarely occur as a primary oncogenic driver and is more frequently identified in the context of acquired bypass resistance to EGFR-TKIs [4, 5]. On the other hand, activating mutations in the MET TKD have only been found in 13–20% of type 1 papillary renal cell carcinomas (pRCC), including V1092I, H1094Y/R/L, H1124D, L1195F/V, F1200I, V1188L, Y1220I, D1228H/N, Y1230C/D/H, M1131T, and M1250T, which result in constitutive ligand-independent MET receptor activation and prompt the downstream oncogenic signalling pathways [6].

In NSCLC patients harboring *METex14* or *METamp*, additional *MET* activating mutations have been clinically documented and preclinically characterized to confer resistance to type I MET inhibitors (i.e., crizotinib, capmatinib and savolitinib) while being sensitive to type II MET inhibitors (i.e., cabozantinib, glesatinib and merestinib) [7–10]. In particular, D1228N/H and Y1230H/C could mediate resistance to crizotinib in *METex14*-altered NSCLC by disrupting drug binding. Recent studies have also shown that mutations in L1195 and F1200 residues confer acquired resistance to type II MET-TKIs [11, 12]. However, previous studies were mainly based on case studies, and a comprehensive analysis of the variety of *MET* TKD mutations that might contribute to acquired resistance to TKIs is required.

In this study, we assessed the targeted sequencing data of 86 NSCLC patients harboring *MET* TKD mutations. Our study revealed a broad spectrum of *MET* TKD mutations and provided insights into potential acquired resistance mechanisms to TKIs.

## Methods

### Patient samples

The extensive database search identified 54,752 NSCLC patients whose tumor specimens and liquid biopsies, including plasma, cardiomyocyte fluid, pleural effusion, and cerebrospinal fluid, were analyzed by targeted NGS between June 2015 and November 2022 at Nanjing Geneseeq Technology Inc. This study included 138 *MET* TKD mutation-positive patients whose samples were collected at all participating hospitals. The study was approved by the Medical Ethics Committee of Nanjing Geneseeq Medical Laboratory (NSJB-MEC-2023-01). Written consent was obtained from each patient before sample collection. Qualified samples were analyzed by targeted next-generation sequencing using targeted gene panels in a CLIA-certified and CAP-accredited clinical testing laboratory (Nanjing Geneseeq Technology Inc., Nanjing, China). Formalin-fixed paraffin-embedded (FFPE) tissue samples were confirmed by pathologists from the centralized clinical testing center before genetic testing. Liquid biopsies were shipped within 48 h of sample collection to the central testing laboratory for cell-free DNA (cfDNA) extraction and the following tests. Clinical characteristics and treatment history were extracted from medical records.

### Targeted next-generation sequencing

DNA extraction was carried out following standard protocols as previously described [13, 14]. Specifically, FFPE samples were de-paraffinized with xylene, followed by genomic DNA extraction using the QIAamp DNA FFPE Tissue Kit (Qiagen Cat. No. 56404) according to the manufacturer’s instructions. Liquid biopsy samples were centrifuged at 1800 g for 10 min, followed by plasma cfDNA extraction and purification using the QIAamp Circulating Nucleic Acid Kit (Qiagen Cat. No. 55114). The cfDNA fragment distribution was analyzed on a Bioanalyzer 2100 using the High Sensitivity DNA Kit (Agilent Technologies, Santa Clara, CA, 5067-4626). As a normal control, the genomic DNA of white blood cells in sediments was extracted using the DNeasy Blood & Tissue Kit (Qiagen Cat. No. 69504). The DNA concentration was quantified using the dsDNA HS assay kit on a Qubit 3.0 fluorometer (Life Technology, US) according to the manufacturer’s recommendations. The NGS library was constructed using the KAPA Hyper Prep kit (KAPA Biosystems) with an optimized manufacturer’s protocol for different sample types. Hybridization-based target enrichment was carried out with GeneseeqPrime™ targeted NGS panel and xGen Lock-down Hybridization and Wash Reagents Kit (Integrated DNA Technologies) [15]. The target-enriched library was then sequenced on HiSeq4000 or HiSeq4000 NGS platforms (Illumina) according to the manufacturer’s instructions.

### Mutation calling

Sequencing data were first demultiplexed and subjected to FASTQ file quality control using Trimmomatic [16]. Qualified data (QC above 15 and without extra N bases) was then subjected to human genome mapping (hg19) using Burrows-Wheeler Aligner (BWA-mem, v0.7.12; https://github.com/lh3/bwa/tree/master/bwakit). Genome Analysis Toolkit (GATK 3.4.0; https://software.broadinstitute.org/gatk/) was employed to perform local realignment around the indels and base quality score recalibration. Picard was used to remove duplicates generated during sample preparation. VarScan2 was applied to detect single-nucleotide variations (SNVs) and insertion/deletion mutations. SNVs were filtered out if the variant allele frequency (VAF) was less than 1% for tumor tissue and 0.3% for liquid biopsy samples. Common SNVs were excluded if they were present in > 1% population in the 1000 Genomes Project or the Exome Aggregation Consortium 65,000 exomes database. The resulting mutation list was further filtered by an in-house list of recurrent artifacts based on a normal pool of whole blood samples. Parallel sequencing of matched white blood cells (control) from each patient was performed to remove sequencing artifacts, germline variants, and clonal hematopoiesis.

### Statistical analysis

Two-proportional t-test was used to determine whether the two proportions were different from each other. Wilcoxon signed-rank test was used to compare if there were differences between paired observations. A two-sided *P* value of less than 0.05 was considered significant for all tests unless indicated otherwise (*P < 0.05, **P < 0.01, ***P < 0.001). All statistical analyses were performed in R (version 4.1.3).

## Results

### Patient overview

**Fig. 1:**
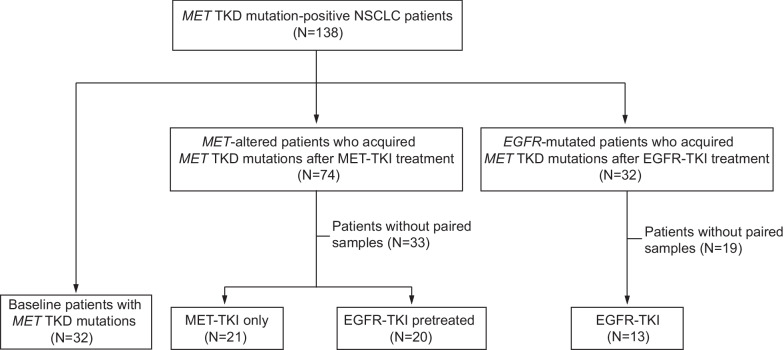
Patient cohorts. A total of 138 NSCLC patients harboring *MET* tyrosine kinase domain (TKD) mutations were included in the study cohort. After excluding patients without paired samples, 86 *MET* TKD mutation-positive patients were included in the final analysis, which was subdivided into three cohorts: baseline (N = 32), MET-TKI-treated (N = 41), and EGFR-TKI-treated (N = 13)

A total of 138 NSCLC patients harboring somatic mutations in the MET TKD assessed by targeted NGS were identified from an extensive database search (Fig. 1). After excluding patients without paired treatment samples, 86 NSCLC patients were included in the final analysis and subdivided into three cohorts depending on the time of detecting *MET* TKD mutations. In particular, *MET* TKD mutations were identified in 32 treatment-naïve NSCLC patients (cohort 1), 41 MET-TKI-treated patients (cohort 2), and 13 EGFR-TKI-treated patients (cohort 3). Notably, 20 patients in cohort 2 were pretreated with EGFR-TKIs before the onset of MET-TKI treatment. The clinical characteristics of these 86 patients are summarized in Table 1.

**Table 1:** Demographics and clinical characteristics of patients (N = 86)

Characteristics	N (%)
Sex	
Female	32 (37.2)
Male	54 (62.8)
Age (years)	
≥ 60	52 (60.5)
< 60	32 (37.2)
Unknown	2 (2.3)
Cohort	
Baseline	32 (37.2)
MET-TKI	41 (47.7)
EGFR-TKI	13 (15.1)
Stage at diagnosis	
I	1 (1.2)
III	1 (1.2)
IV	44 (51.1)
Unknown	40 (46.5)
Histology	
ADC	63 (73.2)
ASC	1 (1.2)
SCC	2 (2.3)
Unknown	20 (23.3)

*ADC* adenocarcinoma, *ASC* adenosquamous carcinoma, *SCC* squamous cell carcinoma

### *MET* kinase domain mutations were identified in baseline NSCLC patients

**Fig. 2:**
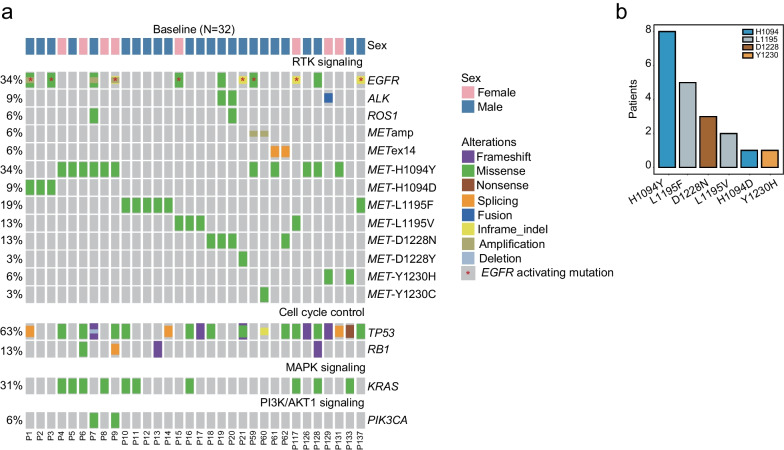
Somatic gene alterations associated with baseline patients. **a **The genomic landscape of *MET* TKD mutation-positive baseline patients (N = 32). Each column represents one patient. The frequency of each gene alteration is listed on the left. **b** The bar graph demonstrates the number of baseline patients who harbor *MET* TKD mutations but no previously reported oncogenic driver alterations

Mutations in MET TKD may lead to ligand-independent receptor phosphorylation and activate downstream oncogenic signaling pathways (Additional file 1: Figure S1). However, oncogenic activation through *MET* TKD mutations has rarely been reported in NSCLC patients. In our study, *MET* TKD mutations were identified in treatment-naïve patients at an extremely low frequency (0.06%, 32/54,752), including H1094Y/D, L1195F/V, D1228N/Y and Y1230C/H (Fig. 2a). Previously reported oncogenic alterations, including *EGFR*-sensitizing mutations (25%, 8/32), *METamp* (6%, 2/32), and *METex14* (6%, 2/32), were identified in these baseline patients. Additionally, one patient exhibited an *ALK* rearrangement, another known oncogenic driver in NSCLC. By comparing the mutational profiles of these patients, we found that 37.5% of baseline *MET* TKD mutation-positive patients (12/32) harbor at least one known oncogenic driver alteration. Of those 20 mutually exclusive *MET* TKD mutations found at baseline, H1094Y was detected in 8 patients, followed by L1195F and D1228N in 5 and 3 individuals, respectively (Fig. 2b). L1195V, H1094D, and Y1230H were also identified in baseline NSCLC patients at a relatively low frequency. The variant allele frequency (VAF) of *MET* TKD mutations in baseline patients was significantly lower than that of *EGFR*-activating mutations (Additional file 2: Figure S2a).

### Second-site *MET* mutations as a general resistance mechanism to MET-TKIs

**Fig. 3:**
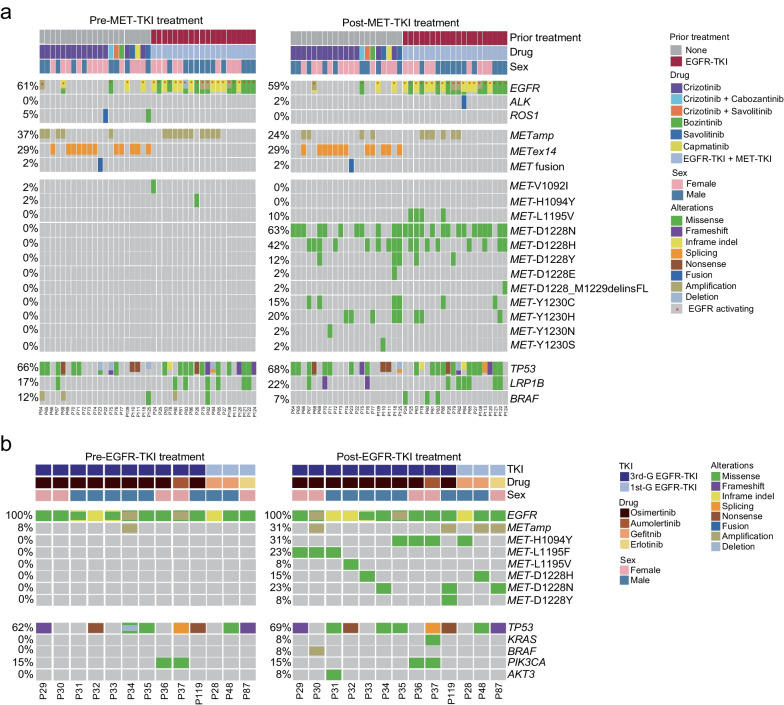
Genomic alterations identified in NSCLC patients at resistance to TKIs. Frequency of genomic alterations in paired samples of MET-TKI-treated (**a**) or EGFR-TKI-treated (**b**) NSCLC patients. Each column represents one patient. The clinical characteristics of patients are shown at the top. The frequency of each gene alteration pre- and post-treatment is listed to the left of the heatmap

*MET* TKD mutations, including D1228N/H and those within codons L1195 and F1200, have been clinically documented and preclinically characterized to confer resistance to MET-TKIs in *METex14*-altered or *MET*-amplified NSCLC patients [9, 12, 17]. Here, we identified 41 patients who acquired additional *MET* TKD mutations after MET-TKI treatment. To understand the implication of these mutations in resistance or sensitivity to MET-TKIs, we performed comprehensive genomic profiling using matched samples before and after TKI treatment. Notably, 20 patients were treated with first-line EGFR-TKIs before the onset of MET-TKI treatment due to activating *EGFR* mutations present at baseline (Fig. 3a). Interestingly, various *MET* TKD mutations were identified in MET-TKI-treated samples, for example, D1228N (63%), D1228H (42%), Y1230H (20%), Y1230C (15%), D1228Y (12%), and L1195V (10%). It is worth noting that a double-mutant within codons D1228 and M1229 (*MET*-D1228_M1229delinsFL) was identified in the post-treatment sample of P124. We also observed that V1092I and H1094Y mutations initially identified in samples after first-line EGFR-TKI treatment of patients 24 (P24) and 26 (P26) were no longer detectable after MET-TKI treatment (Fig. 3a). In addition, both the mutational frequency and the copy number of *METamp* significantly dropped in post-MET-TKI treatment samples (Fig. 3a and Additional file 2: Figure S2b). In contrast, neither the mutational frequency nor the VAF of *METex14* showed significant changes in paired samples before and after MET-TKI treatment (Fig. 3a and Additional file 2: Figure S2c). Our findings suggest that acquired *MET* TKD mutations might confer secondary resistance to MET-TKIs in NSCLC patients, especially in those harboring baseline *METex14* alterations.

### *MET* TKD mutations emerge in NSCLC patients after EGFR-TKI treatment

Although activating mutations, such as L1195V/F and D1228N/H, can cause resistance to MET-TKIs, it is still unclear whether *MET* TKD mutations confer resistance in NSCLC patients independent of MET inhibition. On the other hand, different mechanisms of acquired resistance to EGFR-TKIs have been a matter of intense study in the past few decades. Here, we identified 32 *EGFR*-mutated NSCLC patients who acquired *MET* TKD mutations post-EGFR-TKI treatment (Fig. 1). Only patients with paired samples before and after treatment were included in the following analysis. Various *MET* TKD mutations were identified in post-treatment samples, including H1094Y (31%), L1195F (23%), D1228N (23%), D1228H (15%), D1228Y (8%), and L1195V (8%) (Fig. 3b). None of these *MET* TKD mutations were found in samples pre-EGFR-TKI treatment samples. In addition, we found 4 out of 13 patients harboring concurrent *MET* TKD mutations with *METamp* in post-treatment samples. One of the four patients carried an L1195F mutation, one with a D1228H mutation, one with a D1228N mutation, and the fourth with a D1228N/Y double mutation. Meanwhile, we found that *MET*-H1094Y is unlikely to co-occur with previously assessed acquired mechanisms, including *METamp*, *HER2* amplification [18], *BRAF* mutation [19], and increased expression of the receptor tyrosine kinase AXL [20]. Although patient 37 carried concurrent *KRAS*-G12S (VAF 1.59%) and *PIK3CA*-D1045N (VAF 1.64%), the VAF of *MET*-1094Y was significantly higher (VAF 9.9%). In addition, *PIK3CA*-D1045N (VAF 1.64%) identified in the pre-EGFR-TKI treatment sample has not been annotated based on OncoKB (https://www.oncokb.org/). Despite a likely oncogenic role of *PIK3CA*-C604R in patient 36, this mutation was identified in pre-EGFR-TKI treatment samples of the same patient. While the VAF of *EGFR* mutations dropped after EGFR-TKI treatment, the opposite was observed in *MET* TKD mutations (Additional file 2: Figure S2d). These results suggest that H1094Y might confer an acquired resistance mechanism to EGFR-TKIs.

**Fig. 4:**
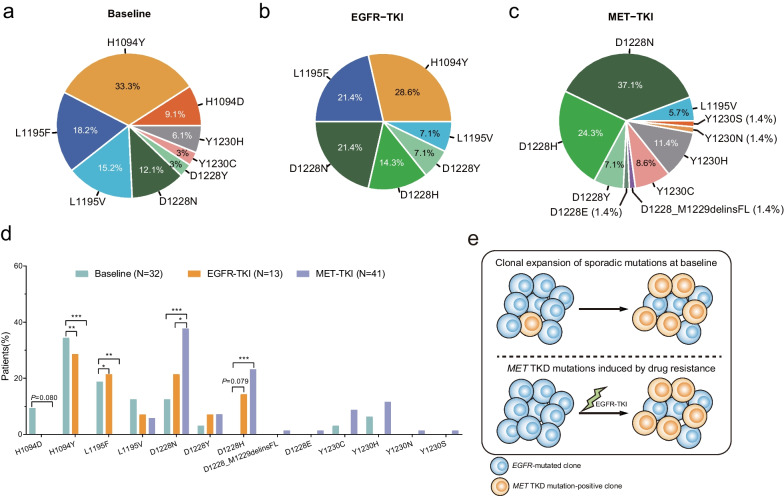
Distribution of *MET* TKD mutations. **a**–**c** Distribution of *MET* TKD mutations in baseline patients (**a**) and patients who acquired *MET* mutations post-EGFR-TKI (**b**) and post-MET-TKI (**c**) treatment. **d** The bar graph demonstrates the proportions of patients in three cohorts harboring each type of *MET* TKD mutation. **e** Schematic demonstration of potential mechanisms of acquiring *MET* TKD mutations after EGFR-TKI treatment

Next, we compared types of *MET* TKD mutations in baseline and TKI-treated cohorts (Fig. 4a–c). Interestingly, *MET*-H1094D was only identified in three baseline patients but not in any of the TKI-treated cohorts (Fig. 4a). However, only patient 2 harbored baseline *MET*-H1094D without any known oncogenic driver mutations (Fig. 2a). At baseline, H1094Y and L1195F were the predominant types and ranked the top two *MET* TKD mutations after excluding known oncogenic drivers, including *EGFR* activating mutations, *METamp*, *METex14*, and *ALK* rearrangement (Figs. 2b and 4a). Besides, *MET*-L1195F mutations, which accounted for 18.2% of all *MET* TKD mutations in baseline patients, were absent from patients who underwent MET-TKI treatment but were found in EGFR-TKI-treated samples (Fig. 4a–c). In contrast, *MET*-L1195V was identified in both MET-TKI and EGFR-TKI treatment cohorts at a lower frequency.

Comparing the two TKI-treated groups, we noticed that H1094Y and L1195F mutations, while being the predominant type of *MET* TKD mutations in the EGFR-TKI group, were absent from the MET-TKI treatment cohort (Fig. 4b–d). However, the high mutational frequency of these mutations at baseline made it difficult to discriminate whether the mutation was induced by clonal expansion of sporadic mutations at baseline or by drug resistance imposed by EGFR inhibitors (Fig. 4e). *MET*-D1228H mutations, on the other hand, showed enrichment in post-EGFR-TKI treatment samples compared to baseline (*P* = 0.079), suggesting a potential acquired resistance mechanism to EGFR-TKIs. In contrast, D1228N and Y1230C/H are more likely associated with acquired resistance to MET-TKIs in NSCLC.

### *MET* TKD mutations are sensitive to combinatorial therapy

Precision medicine using EGFR-TKIs on NSCLC patients harboring *EGFR*-sensitizing mutations has achieved great success. However, disease remission remains a major obstacle due to acquired resistance. Here, we demonstrate that co-targeting both EGFR and MET might be a promising treatment regimen for patients who become resistant to EGFR-TKIs due to acquired *MET* TKD mutations.

**Fig. 5:**
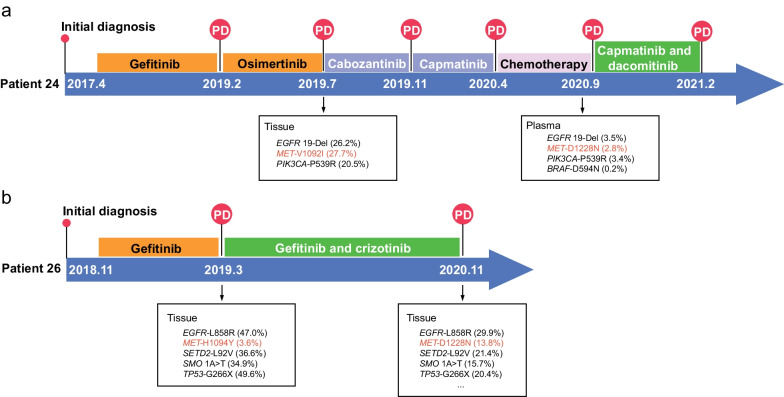
Brief patient case report. The timeline demonstrates that *EGFR*-mutated NSCLC patients benefited from combinatorial therapy with EGFR- and MET-TKIs

In the MET-TKI treatment cohort, V1092I and H1094Y mutations identified in EGFR-TKI pretreated samples that later vanished in post-MET-TKI treatment samples drew our attention (Fig. 3a). Before MET-TKI treatment, P24 underwent first-line gefitinib and second-line osimertinib therapy after the initial diagnosis of stage IV lung adenocarcinoma (Fig. 5a). Owing to the acquisition of *MET*-V1092I mutation (VAF 27.7%), the patient was treated with cabozantinib and capmatinib, followed by two cycles of chemotherapy. Targeted sequencing identified *MET*-D1228N (VAF 2.8%) as well as *PI3KCA* and *BRAF* mutations. Treatment was subsequently switched to combinatorial therapy with capmatinib and dacomitinib, and the patient experienced an overall improvement in the clinical symptoms until the symptoms worsened again after three months of treatment. A similar attempt was made in P26, where treating the patient with gefitinib plus crizotinib effectively overcame the *MET*-H1094Y mutation (Fig. 5b). The patient may benefit from a follow-up treatment with a type II MET-TKI combined with EGFR-TKIs, given that previous studies have demonstrated that the administration of cabozantinib and osimertinib was efficient for rapidly relieving clinical symptoms of patients harboring an acquired mutation of *MET*-D1228N with good tolerance (Table 2) [21]. The potential for sequential use of combinatorial therapy of osimertinib and cabozantinib has also been demonstrated in a case study where a patient with *EGFR*-mutated lung adenocarcinoma acquired four *MET* mutations upon crizotinib treatment [22]. Besides, secondary *MET*-H1094R/Y conferred by osimertinib resistance can be overcome by simultaneous inhibition of MET and EGFR supported by in vitro evidence [23].

**Table 2:** Summary of responses of MET activating mutations to combined targeted therapy

Study	Type	Driver mutation	MET alterations	Treatment	Best response
Kuang et al. [21]	In vivo	EGFR 19-Del	MET-D1228N	Osimertinib + cabozantinib	PR
Kang et al. [22]	In vivo	EGFR-L858R	MET-D1228N/H, MET-Y1230H, MET-D1231Y	Osimertinib + cabozantinib	PD
Schoenfeld et al. [23]	In vitro	EGFR-S768I + V769L	MET-H1094R/Y	Osimertinib + crizotinib	NA

*PR* partial response, *PD* progressive disease, *NA* not available

## Discussion

In this investigation, we delineate mutational profiles of baseline NSCLC patients and paired samples of those who acquired *MET* TKD mutations after TKI treatments. Our research reveals a spectrum of *MET* TKD mutations that might be associated with tumor initiation and drug resistance to TKIs.

In the current era of precision medicine and molecularly targeted therapies, the major task involves finding an optimal treatment choice according to each patient's molecular or genomic characterization of cancer. Evidence has shown that *MET* alterations, including *METex14* mutations and *MET* gene amplification, are primary oncogenic drivers in NSCLC. Sporadic *MET* activating mutations, on the other hand, are only found in about 13–20% of pRCC that result in constitutive activation of MET signaling and prompting the downstream oncogenic signaling cascades. The present study identified *MET* TKD mutations in NSCLC patients before TKI exposure, though at an extremely low frequency. Notably, H1094Y, L1195F/V, and D1228N were among the most frequently found *MET* TKD mutations not concurrent with other known oncogenic driver alterations. Nevertheless, there is currently insufficient evidence suggesting that these *MET* TKD mutations play an oncogenic role in NSCLC. Therefore, considerably more studies are needed before drawing a definitive conclusion.

One significant finding of this study was that a broad spectrum of *MET* TKD mutations was identified in NSCLC patients after MET-TKI and EGFR-TKI treatments. Interestingly, the distribution patterns of these mutations were significantly variable. In *METex14*-altered or *MET*-amplified NSCLC patients, the frequency of D1228N/H mutations was significantly higher than that in baseline and EGFR-TKI-treated patients. 22.8% of all *MET* TKD mutations acquired after MET-TKI treatment were found within codon Y1230. Notably, most of those previously reported *MET* TKD mutations in pRCC have been identified in our study. Of those commonly found mutations in pRCC and NSCLC, V1092I, H1094Y, L1195V, and Y1230H were recognized as oncogenic mutations, while D1228N and Y1230C were considered likely oncogenic alterations based on OncoKB. Furthermore, novel subtype mutations, including D1228Y/E, D1228_M1229delinsFL, and Y1230N/S, whose biological function in tumorigenesis and development is not known, showed a lower frequency than those previously assessed. Despite these mutations being located in the tyrosine kinase domain of MET, whether they function as activating mutations in NSCLC needs further analysis. On the other hand, although *MET*-H1094Y and *MET*-L1195F were found predominantly in patients treated with EGFR-TKI and not in those who underwent MET-TKI treatment, they exhibited high frequency at baseline. Due to the comparable frequency of H1094Y and L1195F in the baseline and EGFR-TKI groups, whether they evolve as subclone mutations or by drug resistance imposed by EGFR-TKIs requires further investigation (Fig. 4e).

Interestingly, *MET*-H1094Y was the most commonly identified *MET* TKD mutation at baseline and the most frequently occurring subtype mutation mutually exclusive to known oncogenic drivers (Fig. 2), implying that it could play an oncogenic role in NSCLC. Consistent with this notion, *MET*-H1094Y is recognized as an oncogenic mutation according to OncoKB. Meanwhile, H1094Y was unlikely to co-occur with other known acquired resistance mechanisms in the EGFR-TKI treatment group (Fig. 3b), suggesting that H1094Y might confer a novel acquired resistance mechanism to EGFR-TKIs. Remarkably, the H1094Y mutation at a VAF of 3.6% identified in patient 26 after first-line EGFR-TKI treatment vanished after second-line MET-TKI treatment, thus, revealing a potential actionable target of MET-TKIs. Moreover, H1094Y might be associated with more effective treatment outcomes of MET-TKIs in *EGFR*-mutated NSCLC patients who acquire this mutation after EGFR-TKI resistance.

On the other hand, *MET*-V1092I was identified at a VAF of 27.7% in patient 24, who underwent first-line EGFR-TKI treatment (Fig. 5a). Since V1092I was undetected at baseline and multiple lines of treatments were applied to the patient after detecting this mutation, whether V1092I is an actionable target of MET-TKI needs further investigation.

There are limitations to our study. Our findings suggest that *EGFR*-mutated NSCLC patients who acquire *MET* TKD mutations could benefit from simultaneous EGFR and MET targeting. However, our argument is potentially compromised by the few cases of combinatorial therapy. Although we listed a few studies from others to address the issue of not having enough patients treated with combinatorial treatment, more in vitro and in vivo studies will contribute to further deciphering the relationship between *MET* TKD mutations and EGFR-TKI resistance. Our extensive database search identified one NSCLC patient harboring a kinase domain deletion mutation of MET after resistance to alectinib, an FDA-approved therapy for treating *ALK*-positive lung cancer. Although we would like to further access *MET* TKD mutations in NSCLC patients who underwent ALK-TKI treatment, the current data from this single patient could not help us gather more information or draw a definitive conclusion. Further investigation using larger cohort samples is warranted.

## Conclusions

Our study evaluates the clinical significance of *MET* TKD mutations in NSCLC. We present substantial evidence suggesting that missense mutations in MET TKD exist at baseline and in patients treated with TKIs. Our findings may provide valuable guidance for clinicians in optimizing treatments for *EGFR*-mutated NSCLC patients harboring *MET* TKD mutations.

## Supplementary Information

**Additional file 1: Figure S1.** Mechanism of MET activation. a MET is the receptor of hepatocyte growth factor (HGF). The α and β chains encompass the remainder of the extracellular domain, the juxtamembrane domain, and the kinase domain. The intracellular part of MET contains a juxtamembrane region responsible for signal regulation and receptor degradation, a catalytic region with enzyme activity, and a C-terminal region acting as a docking site for adaptor proteins. b The binding of HGF to MET leads to receptor dimerization and phosphorylation of tyrosine residues in the kinase domain and autophosphorylation of the C-terminal docking sites, which activates downstream oncogenic signaling pathways, such as RAS/MAPK, PI3K/AKT, and STAT3. AKT, protein kinase B; MET, MET proto-oncogene; PI3K, phosphoinositide 3-kinase; RAS, rat sarcoma virus; MAPK, mitogen-activated protein kinase; STAT, signal transducer and activator of transcription proteins.

**Additional file 2: Figure S2.** Variant allele frequency and copy number change in three patient cohorts. a Variant allele frequency (VAF) of *EGFR* and *MET* TKD mutations in baseline patients. b The copy number of *MET* amplification before and after MET-TKI treatment. c VAF of *MET* exon 14 skipping alterations (*METex14*) before and after MET-TKI treatment. d VAF of *EGFR* and *MET* TKD mutations in patients before and after EGFR-TKI treatment.

## Data Availability

As the study involved human participants, the data cannot be made freely available in the manuscript or a public repository because of ethical restrictions. However, the datasets generated and/or analyzed during this current study are available from the corresponding author upon reasonable request.

## Abbreviations

MET: Mesenchymal epithelial transition factor
*METex14*: *MET* Exon 14 skipping alterations
*METamp*: *MET* Amplification
NSCLC: Non-small-cell lung cancer
TKI: Tyrosine kinase inhibitor
TKD: Tyrosine kinase domain
NGS: Next-generation sequencing
EGFR: Epithelial growth factor receptor
FFPE: Formalin-fixed paraffin-embedded
cfDNA: Cell-free DNA
SNVs: Single-nucleotide variations
VAF: Variant allele frequency
pRCC: Papillary renal cell carcinomas
